# Supplementary material for: Deubiquitinase activity is required for the proteasomal degradation of misfolded cytosolic proteins upon heat-stress
Source: Nat Commun. 2016 Oct 4;7:12907. doi: 10.1038/ncomms12907 (PMC5059457; doi:10.1038/ncomms12907)
Supplement: Supplementary Information — Supplementary Figures 1 – 9 and Supplementary Tables 1 – 2 [file ncomms12907-s1.pdf]

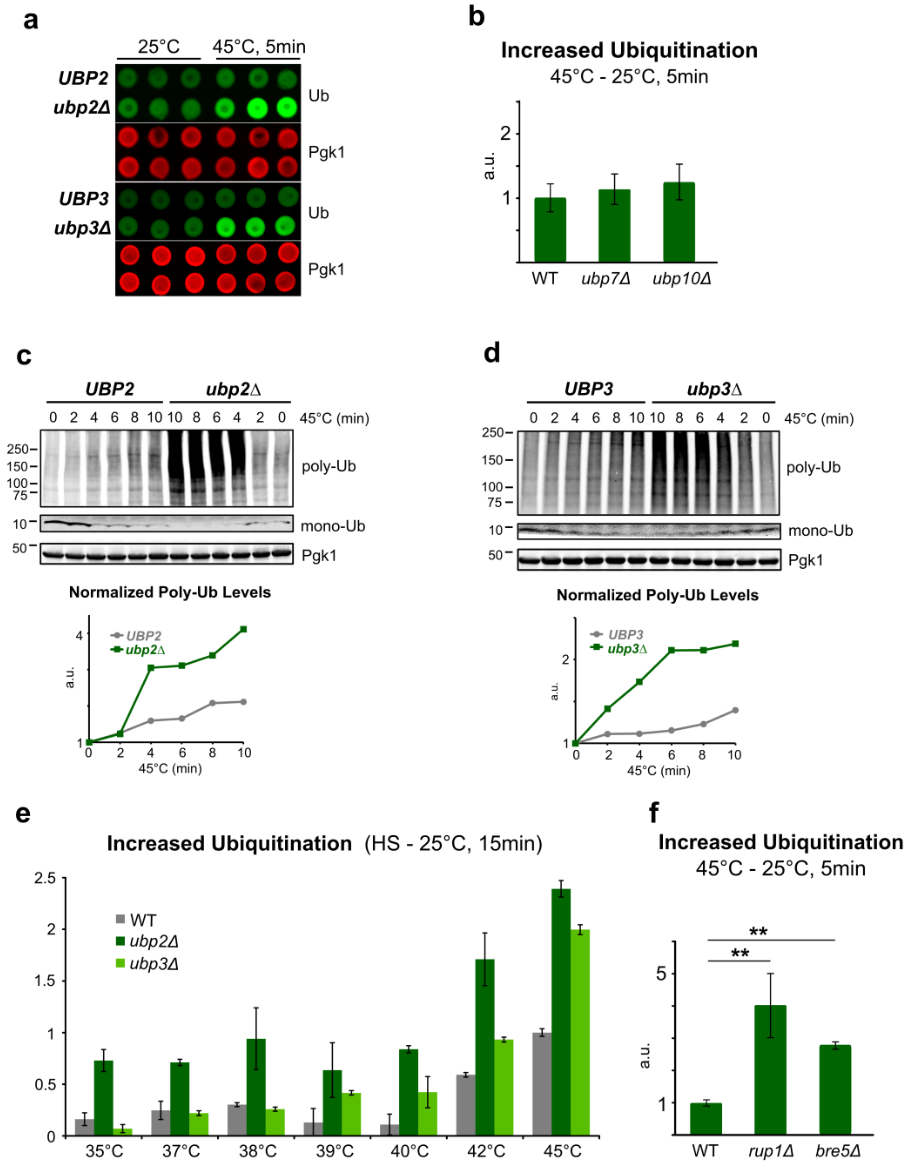


**Supplementary Figure 1. Increased ubiquitination levels in *ubp2*Δ and *ubp3*Δ cells upon HS**
**a.** Dot-blot images for the quantitative data shown in Figure 1B and 1C. **b.** Differences of normalized ubiquitination levels of indicated strains before and after heat-shock quantified by dot-blot. **c-d.** Ubiquitination levels of *ubp2*Δ *(ubp2::HIS3MX6)* or *ubp3*Δ cells compared to wild-type cells at the indicated heat-shock time point were analyzed by Western-blot with anti-ubiquitin antibody. Pgk1 loading-controls and the quantification of poly-ubiquitination levels (above 75 kDa) in each experiment are also shown. **e-f.** Increased ubiquitination levels after a 15 min HS at 45ºC (e) and 5 min HS at 45ºC (f) in the indicated cells quantified by dot-blot and compared to unstressed cells (25°C). In f, increased ubiquitination levels were compared, as shown, to the deletion or WT strains using a two-tail student t-test (***: p<0.001; **: p<0.01; *: p<0.05). For b, e and f, the experiment was done with three biological replicates and averaged values are shown with standard deviations. a.u.: denotes arbitrary units (each value is relative to the reference sample).

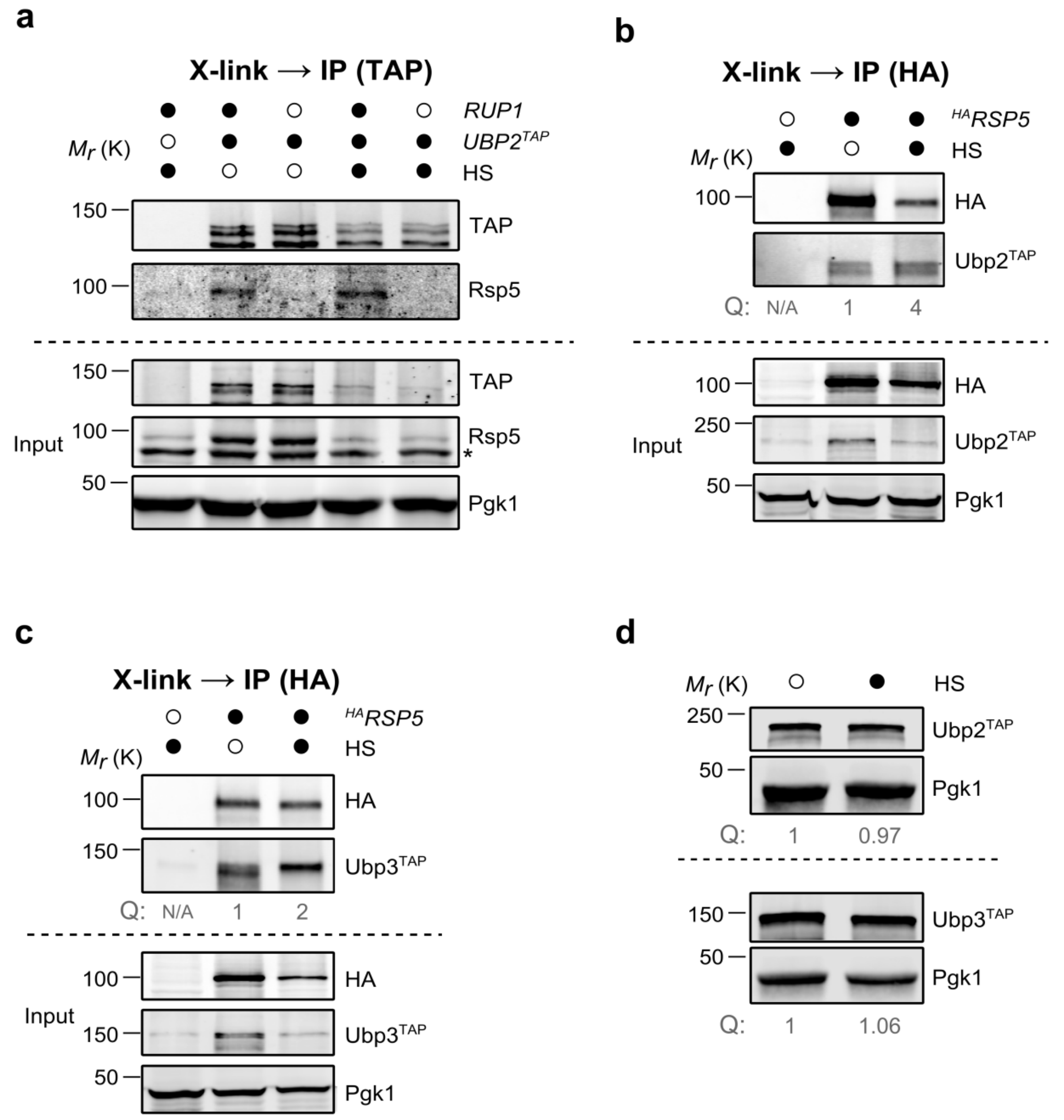


**Supplementary Figure 2. Both Ubp2 and Ubp3 display increased interactions with Rsp5 upon heat-shock**

**a.** Indicated strains that expressed endogenous *RSP5* and C-terminally TAP-tagged *UBP2* were cross-linked for IP experiments. Cells used in all experiments were cross-linked with 1% formaldehyde under no stress or heat-shock (40ºC) conditions for 10min. HS treatments were performed for a total of 20min. Protein levels in both IPs and Inputs are shown. *: denotes unspecific bands detected by the Rsp5 antibody. **b-c.** Cells that expressed endogenously C-terminally TAP-tagged *UBP2* (b) or *UBP3* (c) and carried out a plasmid that expressed 3xHA-tagged Rsp5 or an empty plasmid were subjected to IP experiments. **d.** Cells expressing endogenously C-terminally TAP-tagged *UBP2* or *UBP3* were subjected to HS (40ºC, 20min) or not (25ºC). TAP-Ubp2 or Ubp3 and Pgk1 levels in total cell extracts were analyzed using anti-TAP and anti-Pgk1 antibodies.

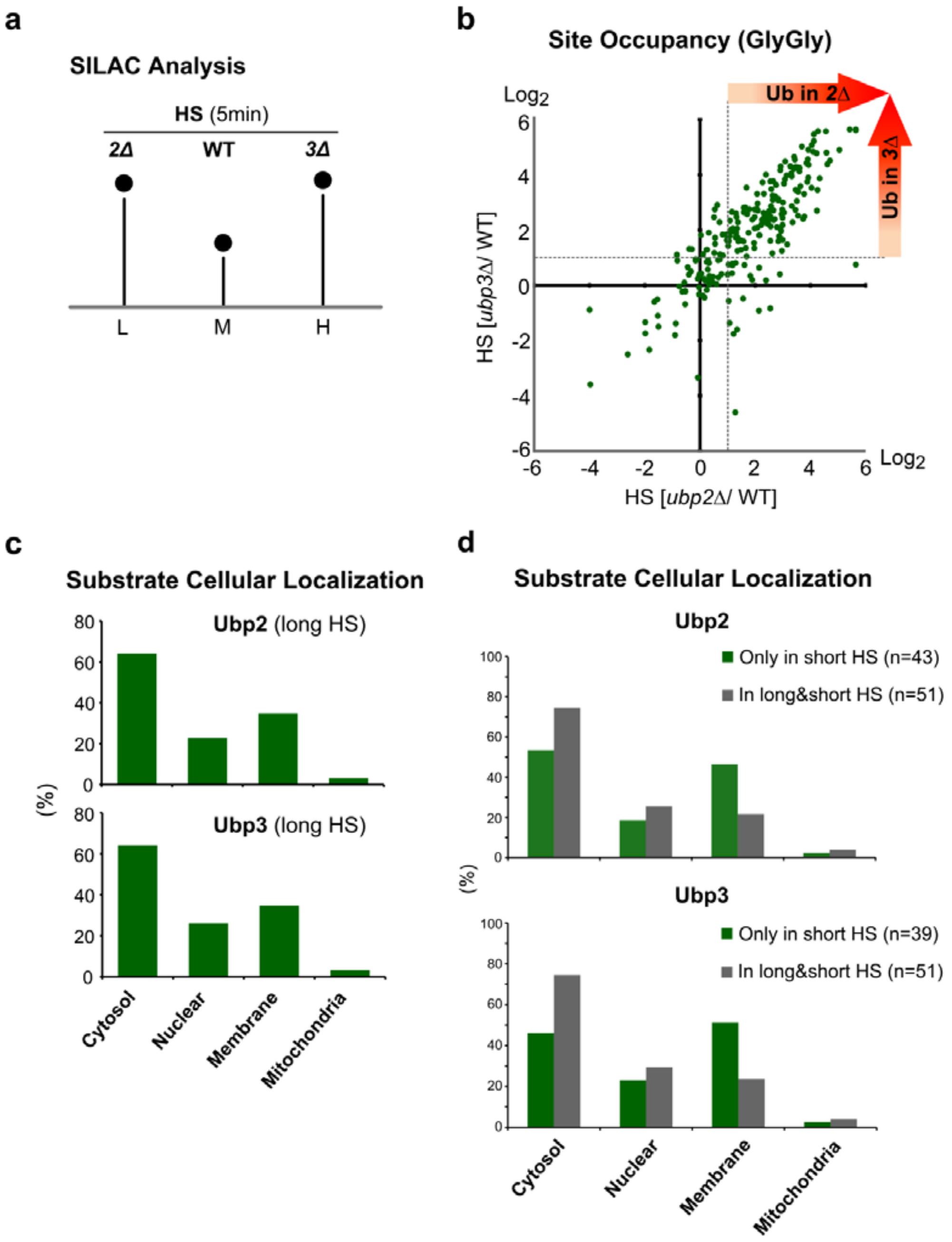


**Supplementary Figure 3. Deletion of *UBP2* or *UBP3* does not alter ubiquitin conjugation site occupancy**

**a.** Schematic representation of the triple SILAC experiment to quantify changes in ubiquitination site occupancy in light-labeled (L) *ubp2*Δ, medium-labeled (M) wild-type and heavy-labeled (H) *ubp3*Δ cells treated by a short heat-shock (45ºC, 5min). **b.** Scatter plots of the $\log_2$ ratios of quantified conjugated peptides (y and x axes represent H/M and L/W ratios, respectively). The red gradient arrows indicate elevated conjugation in *ubp2*Δ or *ubp3*Δ cells upon HS. **c.** Cellular localizations of Ubp2 or Ubp3 candidate substrates upon HS ($\log_2$ ratios $\geq$1) are shown in percentage. **d.** Cellular localizations of Ubp2 or Ubp3 candidate substrates found in short HS dataset only (green) or found shared between short & long HS datasets (gray) are shown in percentage.

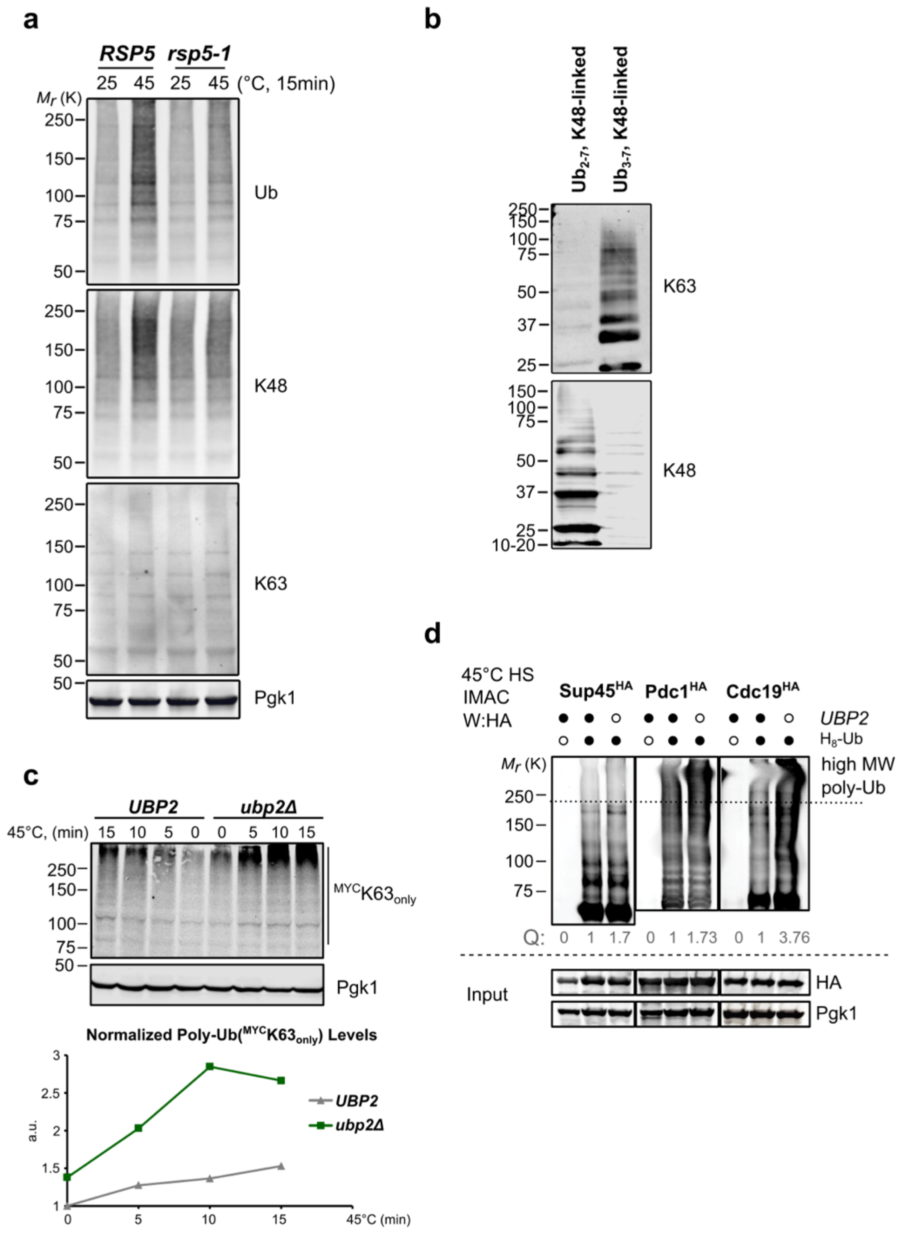


**Supplementary Figure 4. Ubp2 controls ubiquitination levels of Rsp5 heat-induced substrates**

**a.** Levels of K48- and K63-linked ubiquitin in WT and *rsp5-1* cells were assessed using the designated chain specific antibodies in cells grown 25°C and HS at 45°C for 15 min when indicated. **b.** Specificity of anti-K48 linkage and anti-K63 linkage antibodies were tested using poly-ubiquitin chains, 2-7 K48-linked (left) or 3-7 K63-linked (right) from Boston Biochem. **c.** *UBP2* and *ubp2*Δ cells that expressed $^{MYC}$ubiquitin K63-only were incubated at 45ºC for indicated time. Relative anti-Myc signal (>75 kDa) are shown relative to time 0 in *UBP2* cells. **d.** Wild-type (WT) and *ubp2*Δ cells that expressed $H_8$-ubiquitin and endogenously and C-terminally 3xHA-tagged substrates, as indicated, were submitted to a heat-shock (45°C, 20min) prior to the enrichment of conjugated proteins with $Ni^{2+}$ beads and Western-blot with the anti-HA antibody. Inputs and Pgk1 controls are also shown.

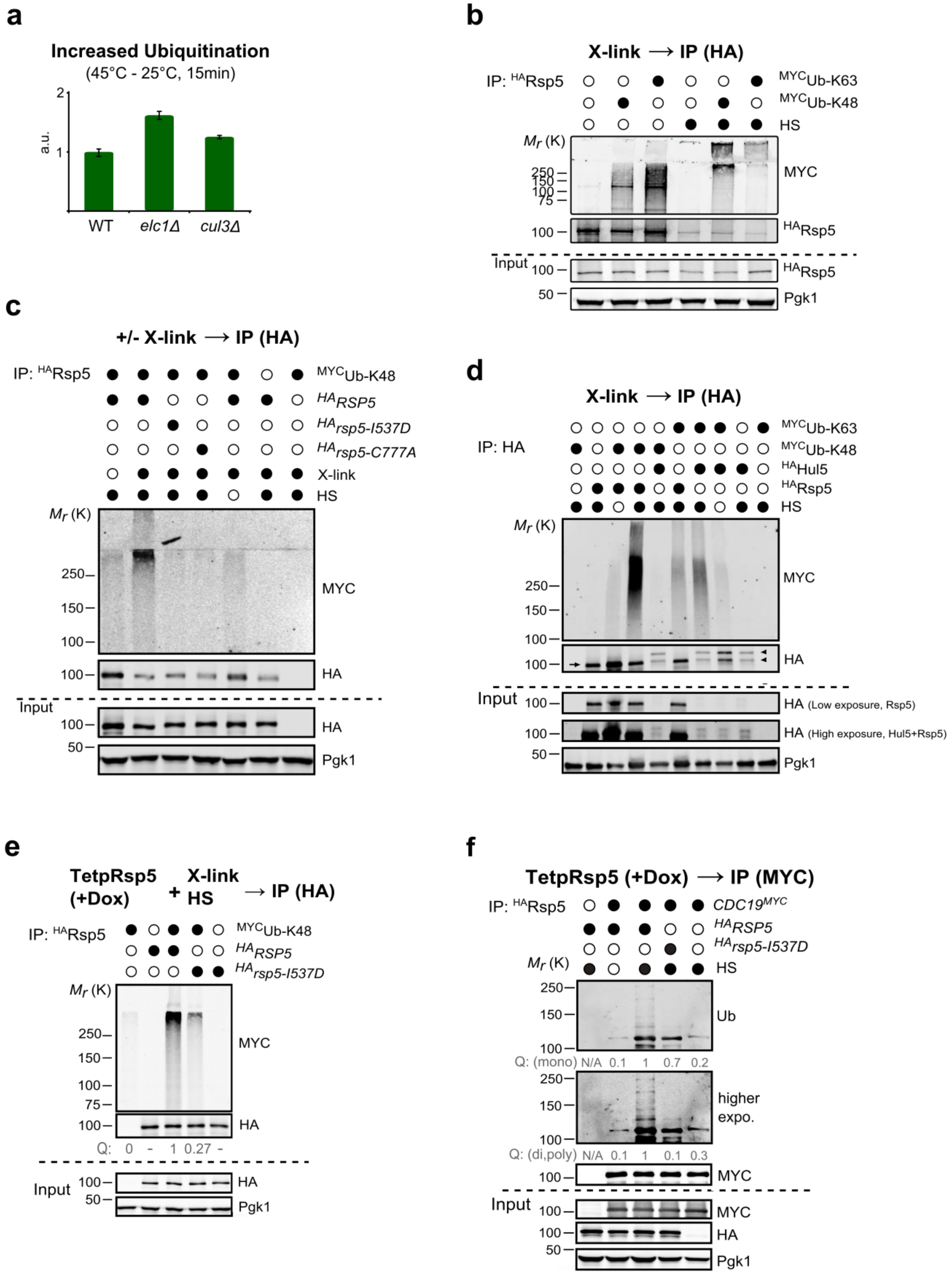

a
Increased Ubiquitination
(45°C - 25°C, 15min)
a.u.
WT
elc1Δ
cul3Δ
b
X-link → IP (HA)
IP: HARsp5
MYCUb-K63
MYCUb-K48
HS
Mr (K)
MYC
HARsp5
Input
Pgk1
c
+/- X-link → IP (HA)
IP: HARsp5
MYCUb-K48
HARSP5
HArsp5-I537D
HArsp5-C777A
X-link
HS
MYC
HA
Input
Pgk1
d
X-link → IP (HA)
IP: HA
MYCUb-K63
MYCUb-K48
HAHul5
HARsp5
HS
MYC
HA
Input
HA (Low exposure, Rsp5)
HA (High exposure, Hul5+Rsp5)
Pgk1
e
TetpRsp5 (+Dox) + X-link HS → IP (HA)
IP: HARsp5
MYCUb-K48
HARSP5
HArsp5-I537D
MYC
HA
Q: 0 - 1 0.27 -
Input
HA
Pgk1
f
TetpRsp5 (+Dox) → IP (MYC)
IP: HARsp5
CDC19MYC
HARSP5
HArsp5-I537D
HS
Ub
Q: (mono) N/A 0.1 1 0.7 0.2
higher expo.
Q: (di,poly) N/A 0.1 1 0.1 0.3
MYC
Input
MYC
HA
Pgk1

**Supplementary Figure 5 (previous page). Upon heat-shock proteins conjugated to K48-chains specifically co-immunoprecipate with Rsp5.**

**a.** Histograms of the differences of the normalized ubiquitination levels of the indicated strains before and after heat-shock (45°C, 15 min) quantified by dot-blot. The experiment was done with three biological replicates and averaged values are shown with standard deviations. a.u.: denotes arbitrary units (each value is relative to the reference sample). **b.** Cells expressing from two plasmids $^{3HA}$Rsp5 and $^{MYC}$Ub-K63 or $^{MYC}$Ub-K48 were cross-linked for 10min with 1% formaldehyde under no stress or heat-shock (40ºC) conditions prior to anti-HA immunoprecipitation (IP). Both protein levels prior (Input) and after IP are shown. **c.** Cells that expressed as indicated $^{3HA}$Rsp5, $^{3HA}$Rsp5-I537D or $^{3HA}$Rsp5-C777A together with empty or $^{MYC}$ubiquitin-K48only constructs were heat-shocked for 20 min (40°C) and cross-linked with 1% formaldehyde for the remaining 10 min prior to lysis with SDS and the anti-HA IP. Controls experiments without the cross-linking or HS were also performed in parallel. Western blot analysis of both IP and Input samples are shown. **d.** Cells expressing from two plasmids either $^{3HA}$Rsp5 or $^{3HA}$Hul5 and $^{MYC}$Ub-K63 or $^{MYC}$Ub-K48 were cross-linked for 10min with 1% formaldehyde under no stress or heat-shock (20min, 40ºC) conditions prior to anti-HA immunoprecipitation (IP). Both protein levels prior (Input) and after IP are shown. Hul5 and Rsp5 are indicated by black arrow heads and arrow respectively. For inputs, HA-Hul5 is shown in a separate panel with higher exposure (below), as it is expressed at lower levels. **e.** Plasmid-expressed Cdc19$^{13MYC}$ was immunoprecipitated with the 9E10 anti-MYC magnetic beads (Origene) from *Tetp::RSP5* cells grown with doxycycline (Dox) that were or not heat-shocked (40ºC, 20min). Cells also expressed from a plasmid $^{3HA}$Rsp5 or $^{3HA}$Rsp5-I537D and were treated with doxycycline to down-regulate endogenous Rsp5 expression. Ubiquitinated Cdc19 was detected using the anti-ubiquitin antibody, and the ratios of both mono- and di, poly-ubiquitination levels relative to wild-type Rsp5 expressing cells are shown. Both protein levels prior (Input) and after IP are shown. **f.** *Tetp::RSP5* cells grown with doxycycline (Dox) and that expressed $^{3HA}$Rsp5 or $^{3HA}$Rsp5-I537D together with empty or $^{MYC}$ubiquitin-K48only constructs were HS (40ºC) for 20 min and cross-linked with 1% formaldehyde for the last 10 min prior to lysis with SDS and IP with anti-HA antibodies and Western blot analysis.

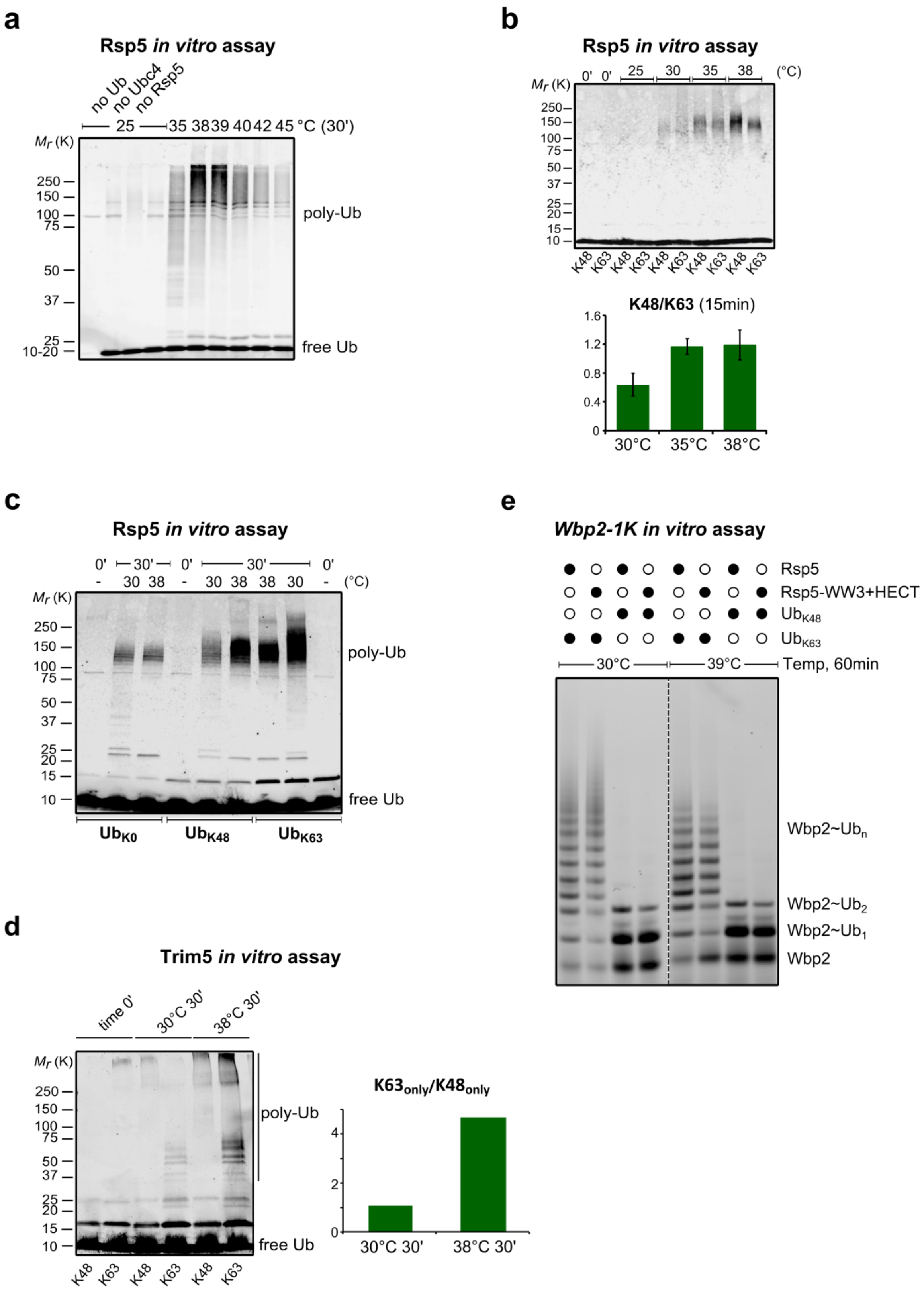

a
Rsp5 in vitro assay
no Ub
no Ubc4
no Rsp5
25 35 38 39 40 42 45 °C (30')
Mr (K)
250
150
100
75
50
37
25
10-20
poly-Ub
free Ub
b
Rsp5 in vitro assay
0' 0' 25 30 35 38 (°C)
Mr (K)
250
150
100
75
50
37
25
20
15
10
K48 K63 K48 K63 K48 K63 K48 K63 K48 K63
K48/K63 (15min)
1.6
1.2
0.8
0.4
0
30°C 35°C 38°C
c
Rsp5 in vitro assay
0' 30' 0' 30' 0'
- 30 38 - 30 38 38 30 - (°C)
Mr (K)
250
150
100
75
50
37
25
20
15
10
poly-Ub
free Ub
Ub$_{K0}$
Ub$_{K48}$
Ub$_{K63}$
d
Trim5 in vitro assay
time 0'
30°C 30'
38°C 30'
Mr (K)
250
150
100
75
50
37
25
20
15
10
poly-Ub
free Ub
K48 K63 K48 K63 K48 K63
K63$_{only}$/K48$_{only}$
4
2
0
30°C 30'
38°C 30'
e
Wbp2-1K in vitro assay
Rsp5
Rsp5-WW3+HECT
Ub$_{K48}$
Ub$_{K63}$
30°C 39°C Temp, 60min
Wbp2~Ub$_n$
Wbp2~Ub$_2$
Wbp2~Ub$_1$
Wbp2

**Supplementary Figure 6 (previous page). Rsp5 can build K48-linkages in the presence of Ubp2 upon heat-shock**

**a.** *In vitro* Rsp5 auto-ubiquitination reactions with Rsp5, Ube1, Ubc4 and $His_6$-ubiquitin (unless specified otherwise) at different temperatures for 30min were analyzed by Western-blot with the anti-ubiquitin antibody. **b.** Same *in vitro* Rsp5 auto-ubiquitination reactions as in Figure 5A but after a 15min incubation. The reactions were carried out with Rsp5, Ube1, Ubc4 and the indicated $His_6$-ubiquitin variant (K63only or K48only) and then analyzed by Western-blot with the anti-ubiquitin antibody. Histograms show the ubiquitination levels in K48only versus K63only experiments averaged from three independent experiments (with standard deviations). Levels were not quantified at 25°C due to the low signal. **c.** *In vitro* Rsp5 auto-ubiquitination assay at the indicated temperatures with $His_6$-tagged lysine-less (K0), K48-only (K48) or K63-only (K63) ubiquitin variants, followed by Western blotting with the anti-ubiquitin antibody (MAB1510). **d.** In vitro auto-ubiquitination reactions with Trim5α, Ube1, Ube2N/Ube2V2 and $His_6$-ubiquitin variant (K63only or K48only) at 30ºC or 38ºC for 30 min were analyzed by Western-blot with the anti-ubiquitin antibody. Histograms show the ubiquitination levels in K48only versus K63only at 30ºC and 38ºC. **e.** *In vitro* ubiquitination reactions at 30 ºC or 39ºC in the presence of fluorescein-conjugated Wbp2-K222only for 60 min. The reactions were carried out with Wbp2, Rsp5 or truncated version of Rsp5 (WW3+HECT), Ube1, Ubc4 and ubiquitin K48only or K63only mutants analyzed by direct visualization of Wbp2 in protein gel.

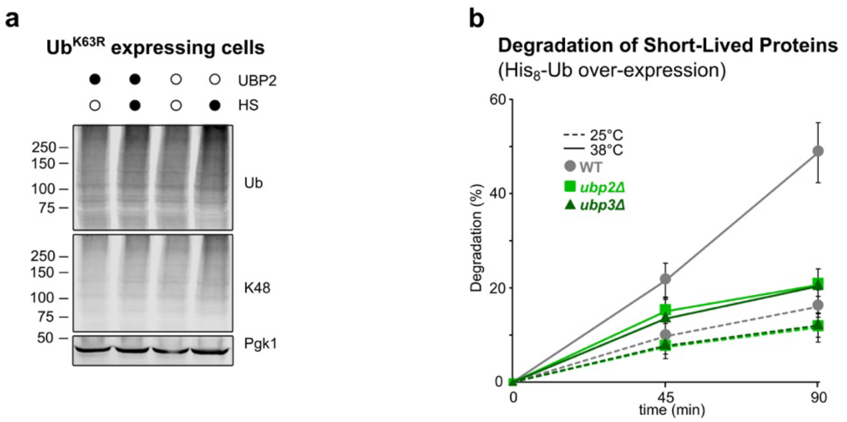


c

**Degradation of Short-Lived Proteins in WT cells**
(His$_8$-Ub varients over-expression)

| Time | 90 mim | |
|---|---|---|
| Temp \ Vector | Ub | Ub$^{K48R}$ |
| 25ºC | 19.0% | 15.0% |
| 38ºC | 62.2% | 45.4% |

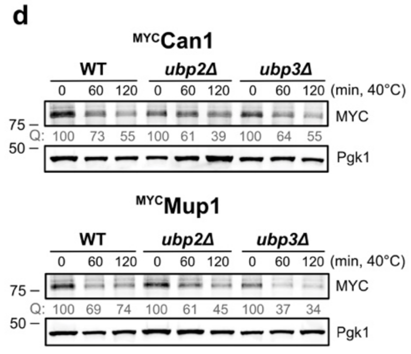


**Supplementary Figure 7. Deletion of *UBP2* or *UBP3* does not affect the turnover of misfolded plasma membrane and aggregation-prone proteins upon heat-shock**

**a.** Levels of total ubiquitination and K48 linkages were assessed in total cell lysates from WT or ubp2Δ cells that solely expressed ubiquitin mutant Ub$_{K63R}$ under heat-shock or no stress conditions. **b.** Degradation of $^{35}$S pulsed-labeled proteins in WT (gray) *ubp2*Δ (light green) and *ubp3*Δ (dark green) cells at 25ºC (dotted lines) or 38ºC (straight lines). The cells expressed His$_8$-ubiquitin from a plasmid (under the *GPD1* promoter) in addition to the endogenous ubiquitin. The portion of proteins degraded at the indicated times was measured and averaged for short-lived proteins in three independent experiments (with standard deviations). **c**. Table shows the degradation rate of $^{35}$S pulsed-labeled proteins in WT cells over-expressing ubiquitin or ubiquitin mutant (Ub$^{K48R}$) at 25ºC or 38ºC for 90 min. **d.** Protein half-life was assessed in WT, *ubp2*Δ and *ubp3*Δ cells that expressed plasmid-encoded $^{13MYC}$Can1 or $^{13MYC}$Mup1 at 40ºC for indicated time after the addition of 100μg/ml cycloheximide. MYC-tagged protein levels were detected using anti-MYC antibody. Pgk1 loading control is also shown.

a

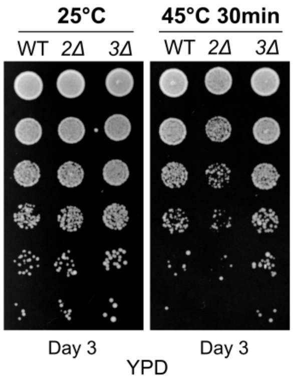


**Supplementary Figure 8. Deletion of *UBP2* or *UBP3* does not affect cell viability upon heat-shock**

**a.** Cell viability was assessed for wild-type (WT), *ubp2*Δ and *ubp3*Δ cells on YPD plates after incubations at 25ºC or 45ºC for 30 min. 1/5 dilutions were used and cells were grown at 25ºC for 3 days.

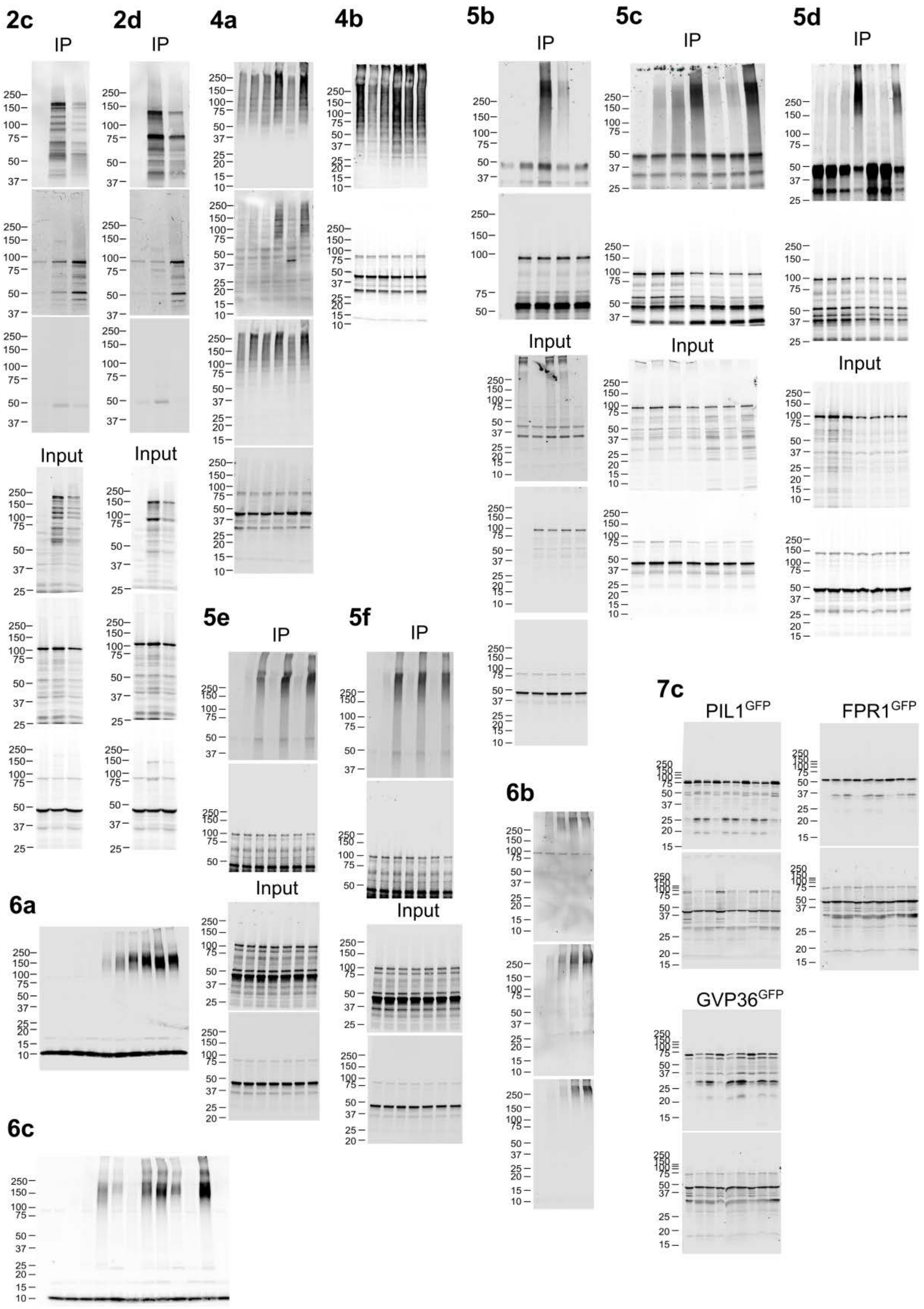


**Supplementary Figure 9. Uncropped images of the most important western blots**
Uncropped images of Western blots from Figure 2c, 2d, 4a, 4b, 5b-f, 6a-c, and 7c are presented with molecular weight ladders.

**Supplementary Table 1.** List of yeast strains used in this study

| Strain ID | Alias | Genotype | Back-ground | Mating Type | Source |
|---|---|---|---|---|---|
| YTM408 | BY4741 | his3Δ1, leu2Δ0, met15Δ0, ura3Δ0 | S288C | a | Open Biosystems |
| YTM409 | BY4742 | his3Δ1, leu2Δ0, lys2Δ0, ura3Δ0 | S288C | alpha | Open Biosystems |
| YTM639 | *rsp5-1* | his3Δ1, leu2Δ, ura3Δ0, met15Δ0, RSP5::*rsp5-1*::$Kan^R$ | S288C | a | C. Boone ts collection |
| YTM884 | ubp2Δ | his3Δ1, leu2Δ0, lys2Δ0, ura3Δ0, ubp2Δ::HIS3MX6 | S288C | alpha | This study |
| YTM1063 | ubp2Δhul5Δ | his3Δ1, leu2Δ0, met15Δ0?, lys2Δ0?, ura3Δ0, ubp2Δ::HIS3MX6, hul5Δ::KanMX6 | S288C | alpha | This study |
| YTM1067 | ubp2Δrsp5-1 | his3Δ1, leu2Δ0, met15Δ0?, lys2Δ0?, ura3Δ0, ubp2Δ::HIS3MX6, RSP5::rsp5-1::KanR | S288C | alpha | This study |
| YTM1076 | ubp1Δ | his3Δ1, leu2Δ0, lys2Δ0, ura3Δ0, ubp1Δ::KanMX6 | S288C | alpha | Open Biosystems |
| YTM1077 | ubp2Δ | his3Δ1, leu2Δ0, lys2Δ0, ura3Δ0, ubp2Δ::KanMX6 | S288C | alpha | Open Biosystems |
| YTM1078 | ubp3Δ | his3Δ1, leu2Δ0, lys2Δ0, ura3Δ0, ubp3Δ::KanMX6 | S288C | alpha | Open Biosystems |
| YTM1079 | ubp4Δ | his3Δ1, leu2Δ0, lys2Δ0, ura3Δ0, ubp4Δ::KanMX6 | S288C | alpha | Open Biosystems |
| YTM1080 | ubp5Δ | his3Δ1, leu2Δ0, lys2Δ0, ura3Δ0, ubp5Δ::KanMX6 | S288C | alpha | Open Biosystems |
| YTM1081 | ubp6Δ | his3Δ1, leu2Δ0, lys2Δ0, ura3Δ0, ubp6Δ::KanMX6 | S288C | alpha | Open Biosystems |
| YTM1082 | ubp7Δ | his3Δ1, leu2Δ0, lys2Δ0, ura3Δ0, ubp7Δ::KanMX6 | S288C | alpha | Open Biosystems |
| YTM1083 | ubp8Δ | his3Δ1, leu2Δ0, lys2Δ0, ura3Δ0, ubp8Δ::KanMX6 | S288C | alpha | Open Biosystems |
| YTM1084 | ub9Δ | his3Δ1, leu2Δ0, lys2Δ0, ura3Δ0, ubp9Δ::KanMX6 | S288C | alpha | Open Biosystems |
| YTM1085 | ubp10Δ | his3Δ1, leu2Δ0, lys2Δ0, ura3Δ0, ubp10Δ::KanMX6 | S288C | alpha | Open Biosystems |
| YTM1086 | ubp11Δ | his3Δ1, leu2Δ0, lys2Δ0, ura3Δ0, ubp11Δ::KanMX6 | S288C | alpha | Open Biosystems |
| YTM1087 | ubp12Δ | his3Δ1, leu2Δ0, lys2Δ0, ura3Δ0, ubp12Δ::KanMX6 | S288C | alpha | Open Biosystems |
| YTM1088 | ubp13Δ | his3Δ1, leu2Δ0, lys2Δ0, ura3Δ0, ubp13Δ::KanMX6 | S288C | alpha | Open Biosystems |
| YTM1089 | ubp14Δ | his3Δ1, leu2Δ0, lys2Δ0, ura3Δ0, ubp14Δ::KanMX6 | S288C | alpha | Open Biosystems |
| YTM1090 | ubp15Δ | his3Δ1, leu2Δ0, lys2Δ0, ura3Δ0, ubp15Δ::KanMX6 | S288C | alpha | Open Biosystems |
| YTM1091 | ubp16Δ | his3Δ1, leu2Δ0, lys2Δ0, ura3Δ0, ubp16Δ::KanMX6 | S288C | alpha | Open Biosystems |
| YTM1092 | yuh1Δ | his3Δ1, leu2Δ0, lys2Δ0, ura3Δ0, yuh1Δ::KanMX6 | S288C | alpha | Open Biosystems |
| YTM1096 | rup1Δ | his3Δ1, leu2Δ0, lys2Δ0, ura3Δ0, rup1Δ::KanMX6 | S288C | alpha | Open Biosystems |
| YTM1098 | bre5Δ | his3Δ1, leu2Δ0, lys2Δ0, ura3Δ0, bre5Δ::KanMX6 | S288C | alpha | Open Biosystems |
| YTM1123 | ubp3Δhul5Δ | his3Δ1, leu2Δ0, met15Δ0?, lys2Δ0?, ura3Δ0, ubp3Δ::KanMX6, hul5Δ::KanMX6 | S288C | a | This study |
| YTM1125 | ubp3Δrsp5-1 | his3Δ1, leu2Δ0, met15Δ0?, lys2Δ0?, ura3Δ0, ubp3Δ::KanMX6, RSP5::rsp5-1::KanR | S288C | alpha | This study |
| YTM1134 | SUP45-3HA | his3Δ1, leu2Δ0, ura3Δ0, MET15, SUP45::SUP45-3HA-HIS3MX6 | S288C | a | This study |
| YTM1295 | PDC1-3HA | his3Δ1, leu2Δ0, lys2Δ0, ura3Δ0, MET15, PDC1::PDC1-3HA::HIS3MX6 | S288C | a | This study |
| YTM1308 | CDC19-3HA | his3Δ1, leu2Δ0, met15Δ0, LYS2, ura3Δ0, CDC19::CDC19-3HA::HIS3MX6 | S288C | a | This study |
| YTM1375 | UBP3-TAP | his3Δ1, leu2Δ0, met15Δ0, ura3Δ0, UBP3::UBP3-TAP-HIS3MX6 | S288C | a | Open Biosystems |
| YTM1481 | UBP2-TAP | his3Δ1, leu2Δ0, met15Δ0, ura3Δ0, UBP2::UBP2-TAP-HIS3MX6 | S288C | a | Open Biosystems |
| YTM1520 | Tetp::RSP5 | his3Δ1, leu2Δ0, met15Δ0, RSP5p::CMV-tTA | S288C | a | Open Biosystems |
| YTM1521 | pCUP1-Ub | lys2-801, leu2-3,112, ura3-52, his3-Δ200, trp1-1, | SUB62 | a | Kindly provided |

| | | | | | |
|---|---|---|---|---|---|
| | | *ubi1::TRP1, ubi2-Δ2::URA3, ubi3-Δub2, ubi4-Δ2::LEU2,pGPD-UBI1, pCUP1-ubi* | | | by Dr. Deshaies, generated by the Finley lab |
| YTM1522 | *pCUP1-Ub-K63R* | *lys2-801, leu2-3,112, ura3-52, his3-Δ200, trp1-1, ubi1::TRP1, ubi2-Δ2::URA3, ubi3-Δub2, ubi4-Δ2::LEU2, pGPD-UBI1, pCUP1-ubiK63R* | SUB62 | a | Kindly provided by Dr. Deshaies, generated by the Finley lab |
| YTM1541 | *pCUP1-Ub/ubp2Δ* | *lys2-801, leu2-3,112, ura3-52, his3-Δ200, trp1-1, ubi1::TRP1, ubi2-Δ2::URA3, ubi3-Δub2, ubi4-Δ2::LEU2,pGPD-UBI1, pCUP1-ubi, ubp2Δ::NatMX4* | SUB62 | a | This study |
| YTM1542 | *pCUP1-Ub-K63R/ubp2Δ* | *lys2-801, leu2-3,112, ura3-52, his3-Δ200, trp1-1, ubi1::TRP1, ubi2-Δ2::URA3, ubi3-Δub2, ubi4-Δ2::LEU2, pGPD-UBI1, pCUP1-ubiK63R, ubp2Δ::NatMX4* | SUB62 | a | This study |
| YTM1558 | *ubp2Δubp3Δ* (1) | *his3Δ1, leu2Δ0, MET15, LYS2, ura3Δ0, ubp2Δ::HIS3MX6, ubp3Δ::KanMX6* | S288C | alpha | This study |
| YTM1559 | *ubp2Δubp3Δ* (2) | *his3Δ1, leu2Δ0, MET15, LYS2, ura3Δ0, ubp2Δ::HIS3MX6, ubp3Δ::KanMX6* | S288C | alpha | This study |
| YTM1592 | *otu1Δ* | *his3Δ1, leu2Δ0, lys2Δ0, ura3Δ0, otu1Δ::KanMX6* | S288C | alpha | Open Biosystems |
| YTM1594 | *otu2Δ* | *his3Δ1, leu2Δ0, lys2Δ0, ura3Δ0, otu2Δ::KanMX6* | S288C | alpha | Open Biosystems |
| YTM1597 | *UBP2-TAP/rup1Δ* | *his3Δ1, leu2Δ0, lys2Δ0?, met15Δ0?, ura3Δ0, UBP2::UBP2-TAP-HIS3MX6, rup1Δ::KanMX6* | S288C | a | This study |
| YTM1600 | *PIL1-GFP* | *his3Δ1, leu2Δ0, met15Δ0, ura3Δ0, PIL1::PIL1-GFP::HIS3MX6* | S288C | a | Open Biosystems |
| YTM1601 | *PIL1-GFP/ubp2Δ* | *his3Δ1, leu2Δ0, LYS2, MET15, ura3Δ0, PIL1::PIL1-GFP::HIS3MX6, ubp2Δ::KanMX6* | S288C | a | Open Biosystems |
| YTM1602 | *PIL1-GFP/ubp3Δ* | *his3Δ1, leu2Δ0, LYS2, MET15, ura3Δ0, PIL1::PIL1-GFP::HIS3MX6, ubp3Δ::KanMX6* | S288C | a | Open Biosystems |
| YTM1603 | *GVP36-GFP* | *his3Δ1, leu2Δ0, met15Δ0, ura3Δ0, GVP36::GVP36-GFP-HIS3MX6* | S288C | a | Open Biosystems |
| YTM1604 | *GVP36-GFP/ubp2Δ* | *his3Δ1, leu2Δ0, LYS2, met15Δ0, ura3Δ0, GVP36::GVP36-GFP-HIS3MX6, ubp2Δ::KanMX6* | S288C | a | Open Biosystems |
| YTM1605 | *GVP36-GFP/ubp3Δ* | *his3Δ1, leu2Δ0, lys2Δ0, MET15, ura3Δ0, GVP36::GVP36-GFP-HIS3MX6, ubp3Δ::KanMX6* | S288C | a | Open Biosystems |
| YTM1606 | *FPR1-GFP* | *his3Δ1, leu2Δ0, met15Δ0, ura3Δ0, FPR1::FPR1-GFP-HIS3MX6* | S288C | a | Open Biosystems |
| YTM1607 | *FPR1-GFP/ubp2Δ* | *his3Δ1, leu2Δ0, lys2Δ0, MET15, ura3Δ0, FPR1::FPR1-GFP-HIS3MX6, ubp2Δ::KanMX6* | S288C | a | Open Biosystems |
| YTM1608 | *FPR1-GFP/ubp3Δ* | *his3Δ1, leu2Δ0, LYS2, MET15, ura3Δ0, FPR1::FPR1-GFP-HIS3MX6, ubp3Δ::KanMX6* | S288C | a | Open Biosystems |

**Supplementary Table 2.** List of plasmids used in this study

| Lab ID | Name | Source & Note | Reference |
|---|---|---|---|
| BPM30 | pRS316-$H_8$-ubiquitin | H8-ubiquitin with GPD promotor and PGK terminator is insert into pRS316 yeast URA3, CEN, ARS vector in the EcoRI and SmaI sites | Mayor, T. *et al.* (2007) MCP 6(11):1885-95 |
| BPM42 | pRS316 | yeast URA3, CEN, ARS vector with MCS. | Sikorski, R. S. and Hieter, P. (1989) Genetics 122:19-27 |
| BPM45 | pRS313 | yeast HIS3, CEN, ARS vector with MCS. | Sikorski, R. S. and Hieter, P. (1989) Genetics 122:19-27 |
| BPM49 | pRS315 | yeast LUE2, CEN, ARS vector with MCS. | Sikorski, R. S. and Hieter, P. (1989) Genetics 122:19-27 |
| BPM98 | GST-Rsp5 | N-terminal GST tag. Ampicillin and chloramphenicol resistance markers. In bacterial strain BL21. | Saeki, Y. (2005) Meth Enz 399:215-27 |
| BPM173 | pRS313-GPD-13myc-PGK | yeast HIS3, CEN/ARS vector. Ampicillin resistance. | Khosrow-Khavar *et al.* (2012) G3 2(5):619-28 |

| | | | |
|---|---|---|---|
| BPM486 | YCplac111-3HA-RSP5 | yeast LUE2, ARS1-CEN4 vector with MCS. Rsp5 with endogenous promoter was cloned in using EcoRI and SphI | B. Gajewska *et al.* (2000) Genetics 157:91-101 |
| BPM552 | pRS416-MUP1-GFP | CAN1 with endogenous promoter and C-terminal GFP tag was cloned in pRS416 | Lin C. H. *et al.* (2008) Cell 135(4):714-25 |
| BPM553 | pRS416-CAN1-GFP | MUP1 with endogenous promoter and C-terminal GFP tag was cloned in pRS416 | Lin C. H. *et al.* (2008) Cell 135(4):714-25 |
| BPM563 | pRS313-GPD-CDC19-13MYC-PGK | Cdc19 was PCR amplified from genomic DNA and inserted into BPM173 using BamHI and XmaI sites. | N.N.Fang *et al.* (2014) NCB (12):1227-37 |
| BPM591 | pRS313-GPD-Myc-Ub-K48only-PGK | Plasmid was created by site directed mutagenesis using BPM593 as a template. Mutations changed R48 to lysine. | N.N.Fang *et al.* (2014) NCB (12):1227-37 |
| BPM592 | pRS313-GPD-Myc-Ub-K63only-PGK | Plasmid was created by site directed mutagenesis using BPM593 as a template. Mutations changed R63 to lysine. | N.N.Fang *et al.* (2014) NCB (12):1227-37 |
| BPM595 | pRS313-GPD-13myc-CAN1-PGK | Plasmid was created by PCR amplification of the ORFs CAN1 from BPM553 and inserted into BPM173 using the NotI site | This study |
| BPM596 | pRS313-GPD-13myc-MUP1-PGK | Plasmid was created by PCR amplification of the ORFs MUP1 from BPM552 and inserted into BPM173 using the NotI site | This study |
| BPM605 | pRS315-UBP2 | Plasmid was generated by inserting PCR amplified UBP2 from genomic DNA (open reading frame with 500bp up and downstream sequences) into pRS315 plasmid using XmaI and SpeI restriction sites | This study |
| BPM606 | pRS315-UBP2(C745A) | Plasmid was created by site directed mutagenesis using BPM605 as a template. Mutations changed C745 to A. | This study |
| BPM607 | pRS315-UBP3 | Plasmid was generated by inserting PCR amplified UBP3 from genomic DNA (open reading frame with 500bp up and downstream sequences) into pRS315 plasmid using XmaI and SpeI restriction sites | This study |
| BPM608 | pRS315-UBP3(C469A) | Plasmid was created by site directed mutagenesis using BPM605 as a template. Mutations changed C469 to A. | This study |
| BPM625 | pRS313-GPD-CDC19(D367R)-13Myc-PGK | Plasmid was created by site directed mutagenesis using BPM563 as a template. Mutations changed D367 to an arginine residue. | N.N.Fang *et al.* (2014) NCB (12):1227-37 |
| BPM740 | GST-UBP2 | N-terminal GST tag in pGEX-6p-1 vector. Ampicillin resistance markers. In bacterial strain BL21. | Lee Y. *et al.* (2005) EMBO J. 24 (13):2414-24 |
| BPM741 | GST-RUP1 | N-terminal GST tag in pGEX-6p-1 vector. Ampicillin resistance markers. In bacterial strain BL21. | Lee Y. *et al.* (2005) EMBO J. 24 (13):2414-24 |
| BPM746 | GST-UBC4 | N-terminal GST tag in pGEX-6p-1 vector. Ampicillin resistance markers. In bacterial strain BL21. | Kim H. C. and Huibregtse J. M. (2009) MCB (12):3307-18 |
| BPM752 | pRS316-5'UTR-Hul5-3'UTR | Plasmid was generated by inserting PCR amplified HUL5 from genomic DNA (open reading frame with 500bp up and 400bp downstream sequences) into pRS316 plasmid using SacII and XhoI restriction sites | This study |
| BPM762 | YCplac111-3HA-RSP5-I537D | Plasmid was created by site directed mutagenesis using BPM486 as a template. Mutations changed I537 to D. | This study |
| BPM782 | YCplac111 | Plasmid was generated by removing Rsp5 ORF using HindIII restriction site for religation | This study |
| BPM812 | pRS316-5'UTR-3HA-HUL5-3'UTR | Plasmid was generated by inserting 3HA sequence amplified from pFA6a-3HA-His3MX6 plasmid into BPM 752 | This study |
| BPM835 | YCplac111-3HA-RSP5-C777A | Plasmid was created by site directed mutagenesis using BPM486 as a template. Mutations changed C777 to A. | This study |
| BPM910 | GST-TRIM5α (RING1-93) | N-terminal GST tag in pGEX-6p-1 vector. Ampicillin resistance markers. In bacterial strain BL21. | Yudina Z. *et al.* (2015) Cell Rep. 12(5):788-97 |
